# Targeting potassium channels for increasing delivery of imaging agents and therapeutics to brain tumors

**DOI:** 10.3389/fphar.2013.00062

**Published:** 2013-05-29

**Authors:** Divya Khaitan, Nagendra S. Ningaraj

**Affiliations:** Department of Molecular Oncology Research, Scintilla Academy for Applied Sciences’ Research and EducationBangalore, Karnataka, India

**Keywords:** blood–brain barrier, blood–brain tumor barrier, drug delivery, brain tumors, metastatic brain tumor, potassium channels, DCE-MRI, biochemical modulation of BTB

## Abstract

Every year in the US, 20,000 new primary and nearly 200,000 metastatic brain tumor cases are reported. The cerebral microvessels/capillaries that form the blood–brain barrier not only protect the brain from toxic agents in the blood but also pose a significant hindrance to the delivery of small and large therapeutic molecules. Different strategies have been employed to circumvent the physiological barrier posed by blood–brain tumor barrier (BTB). Studies in our laboratory have identified significant differences in the expression levels of certain genes and proteins between normal and brain tumor capillary endothelial cells (ECs). In this study, we validated the non-invasive and clinically relevant dynamic contrast enhancing-magnetic resonance imaging (DCE-MRI) method with invasive, clinically irrelevant but highly accurate quantitative autoradiography method using rat glioma model. We also showed that DCE-MRI metric of tissue vessel perfusion-permeability is sensitive to changes in blood vessel permeability following administration of calcium-activated potassium (BK_Ca_) channel activator NS-1619. Our results show that human gliomas and brain tumor ECs that overexpress BK_Ca_ channels can be targeted for increased BTB permeability for MRI enhancing agents to brain tumors. We conclude that monitoring the outcome of increased MRI enhancing agents’ delivery to microsatellites and leading tumor edges in glioma patients would lead to beneficial clinical outcome.

## INTRODUCTION

A significant number of primary tumor cases are reported each year in the US, and the metastatic tumors from systemic cancers are at least 10-fold higher than the primary brain tumors. The magnitude of the problem at the global level is even more staggering. Estimates show that about 50% of patients receiving brain radiation and/or surgical resection have recurrences in the brain within a year, severely shortening life expectancy (Ningaraj, 2006). Targeting gliomas is extremely difficult because brain provides a “safe haven” for tumor cells. The cerebral microvessels/capillaries that form the blood–brain barrier (BBB) block the delivery of most small and large therapeutic molecules (Pardridge, 2003). Different strategies have been employed to circumvent the physiological barrier posed by blood–brain tumor barrier (BTB), often based on a conception of the barrier being controlled by the “neurovascular unit” consisting of endothelial cells (ECs), tight junctional proteins connecting the ECs, glial, pericytes, and astrocytic foot processes, all of which interact with neurons (Quencer and Neuwelt, 2002; Toda, 2003; Zhang et al., 2003; Pardridge, 2004; Rautioa and Chikhale, 2004; Rich and Bigner, 2004; Kinoshita et al., 2006; Lee et al., 2006). However, research should be focused on individual component of the neurovascular unit. The focus is now on targeted cancer therapy by supplementing conventional chemotherapy and radiotherapy with monoclonal antibodies (MAbs; Checkley et al., 2003; Rich and Bigner, 2004; Khaitan et al., 2006; Buie and Valgus, 2008; Mathieu et al., 2008). The purpose of antibody treatment of cancer is to induce the direct or indirect destruction of cancer cells, either by specifically targeting either the tumor or the tumor vasculature (Checkley et al., 2003; Buie and Valgus, 2008). Targeting tumor and tumor blood vessel-specific marker(s) is a good strategy to control tumor growth (Ningaraj et al., 2003a). It is, however, critical to study whether tumor-specific drug delivery has the potential to minimize toxicity to normal tissues, and improve bioavailability of cytotoxic agents to neoplasms. Although the BTB is “leaky” in the tumor center, the established microvessels feeding the proliferating glioma edge as well as the brain tissue surrounding the tumor are nearly as impermeable as the BBB (Inamura and Black, 1994; Liu et al., 2001, 2002; Ningaraj et al., 2002, 2003a, b, 2009a, b; Black and Ningaraj, 2004, 2006, 2007; Khaitan et al., 2009). It is incorrect to assume that the disrupted BBB facilitates drug delivery to gliomas because diffuse tumor cell invasion is a hallmark of even low-grade gliomas.

Drug delivery research focuses on several innovative methods, including nanoparticles, microparticles as carriers of anticancer agents, polyethylene glycol (PEG) technology, encapsulating anticancer drugs in liposomes, and MAbs for the delivery of anticancer payloads (Ningaraj et al., 2007). One area of research has focused on brain microvascular ECs (BVEC), which are a major component of the neurovascular unit. However, many issues that are related to BVEC are still not well understood, including gene and protein profiling in normal brain and brain tumor capillary ECs (Cucullo et al., 2003; Doubrovin et al., 2003; Demeule et al., 2004; Kemper et al., 2004; Jaeger et al., 2005). Research in this field is hampered due to the complexities that are involved in isolating pure BVEC devoid of pericytes, neurons, and tumor cell populations, as well as due to differences between and within brain tumors. For instance, significant differences were found between normal human brain and brain tumor capillaries, including differential expression of large conductance calcium-activated potassium (BK_Ca_) channels (Ningaraj et al., 2002, 2003a; Sontheimer, 2004; Black and Ningaraj, 2006, 2007; Ningaraj, 2006; Khaitan et al., 2009). Recent progress in the molecular targeting of tumor-specific antigens with specific agents, however, can be exploited by identifying additional novel targets for modulating BTB permeability. Studies in our laboratory are investigating whether any significant differences exist in the expression levels of certain genes and proteins or presence of unique molecules in brain tumor capillary ECs that can be modified for increased anticancer drug delivery to brain tumors (Ningaraj et al., 2002, 2003a, b; Black and Ningaraj, 2006, 2007; Khaitan et al., 2009).

The amount of drug that reaches at the tumor site depends on BTB permeability, which varies considerably among brain tumor patients (Fortin et al., 2005). Drug concentrations in brain tissue usually drop with increasing distance from the tumor core, and thus the drug concentration is fairly low in the peripheral parts of the tumor, where tumor cells infiltrate the normal brain. In these areas, where tumor proliferation is most rapid, the BTB is relatively intact (Rice et al., 2003; Ningaraj, 2006). Novel approaches for effective delivery through the BTB of anticancer agents that circumvent active efflux transporters at the BTB, like P-glycoprotein (Pgp)-mediated efflux (Bronger et al., 2005) would provide neuro-oncologists with effective anticancer agents for the effective treatment of gliomas thereby increasing patients’ survival rates. It is known that the BTB is leaky and MRI agents can penetrate easily, however, the BTB is disrupted only when the tumor reaches 1 mm in size. This provides an opportunity for the micro metastatic tumors to survive and spread further. It has been shown that approximately one-third of patients operated for gliomas probably have microsatellites distant from primary tumor core, despite MRI with gadolinium enhancement being the most sensitive imaging modality for both diagnosis and follow-up of patients with gliomas (Checkley et al., 2003; Yankeelov et al., 2005a). In addition, we have validated dynamic contrast enhancing-magnetic resonance imaging (DCE-MRI) method to measure changes in permeability following administration of BTB permeabilizing agent that significantly increases the delivery of gadolinium diethylenetriamine penta-acetic acid (Gd-DTPA) to detect tumor microsatellites and diffused gliomas (Ningaraj, 2006). Such a validated MRI method would significantly influence the medical care of glioma patients by providing valuable information about the microsatellites and diffused glioma boundary that may otherwise go undetected. The improved possibility of quantitating the BTB defect by MRI may give new information about tumor pathogenesis or etiology, leading to improved methods in monitoring the efficacy of treatments in glioma patients.

## MATERIALS AND METHODS

### CELL LINES

Established cell line representing rat glioma (C6) and mouse gliomas (GL26) obtained from American Type Culture Collection were used to study the role of BK_Ca_ channels in BTB permeability regulation. The cancer cells were maintained in minimum essential medium (MEM; Invitrogen, Carlsbad, USA) containing 10% fetal bovine serum (Invitrogen, Carlsbad, USA).

### ANIMAL STUDY APPROVALS

Animal study approvals were obtained from Vanderbilt Ingram Cancer Center-Institutional Animal Care and Use Committee. The animals were housed at the vivarium facility where tumor implantation procedure was carried out. For MRI scanning, the mice and rats were transported to the MRI facility in the adjacent building.

### INTRACRANIAL TUMOR IMPLANTATION

Nine female Wistar rats weighing 200–250 g had 2 × 10^5^ C6 glioma cells injected into the basal ganglia. To study increased delivery of MRI contrast enhancing agent, we used mouse xenograft brain tumor model. Intracranial tumor implantation was accomplished by a stereotactic technique as described in our previous studies (Ningaraj et al., 2002, 2003a, b; Black and Ningaraj, 2006, 2007; Khaitan et al., 2009). Seven days after tumor implantation, the rats were imaged using a Varian 4.7 T scanner equipped with a 63-mm quadrature birdcage coil.

### PREPARATION OF RATS FOR DCE-MRI SCANNING AND T1 MEASUREMENT

All imaging was performed on the 9.4 T/20 cm horizontal bore scanner (Bruker, Billerica, MA, USA) with actively shielded gradients (200 mT/m). Radiofrequency (RF) coils employed was a 35-mm in ID birdcage resonator. Rat was initially anesthetized with 5% isoflurane, which was reduced to 1.5~2% for maintenance during the experiments, and a catheter inserted in the tail vein for administration of saline, Gd-DTPA, and 1,3-dihydro-1-[2-hydroxy-5-(trifluoromethyl) phenyl]-5-(trifluoromethyl)-2H-benzimidazol-2-one (NS-1619). Rat was secured in a head holder with ear bars and a bite bar to prevent head motion, and placed on a hot water pad to maintain body temperature. Rectal temperature, heart rate, and blood oxygenation was continuously monitored during the experiment. Using a three-plane scout sequence, the central imaging slice was placed to view the largest lateral extent of the tumor. For anatomic imaging, T_2_-weighted multi-slice-multi-echo (MSME) method was carried out [repetition time (TR) = 4000 ms; echo time (TE) = 8.6 ms; field of view (FOV) = 30 mm × 30 mm; matrix size = 128 × 128] with 16 slices through the tumor (in-plan voxel dimension = 0.23 mm × 0.23 mm × 1 mm). T_1_ map was acquired with an inversion recovery (IR) true-fast imaging with steady state precession (FISP) method (TR = 2.85 ms; TE = 1.43 ms; FOV = 30 mm × 30 mm; matrix size = 128 × 128; thickness = 1 mm; flip angle (α) = 30°; image frame = 16; range of inversion time (TI) = 100–2564 ms; TI increment = 154 ms). Using the fast automatic shimming technique by mapping along projections (FASTMAP) auto-shimming method, banding artifacts, which occasionally appeared in true-FISP images, was removed by local B_0_ shimming. A DCE-MRI experiment was performed in glioma rat model with a contrast agent (Gd-DTPA). Imaging parameters for the IR true-FISP was as described above except two slices: one was in the largest lateral extent of the tumor and the other in the rat neck for the measurement of the vascular input function (VIF). The VIF was measured in one or both jugular veins in the rat neck. The IR true-FISP block was repeated 80 times. Following eight baseline scans, the contrast agent mixture was administered over 5 min via an infusion pump connected to cannulated tail vein, and immediately followed by a bolus of 1 ml of saline. For BTB permeability assay potassium channel activator (NS1619) was administered via tail vein catheter followed by Gd-DTPA over 5 min through an infusion pump.

### UNILATERAL TRANSFER CONSTANT (Ki) MEASUREMENT

After a wash period of 24 h, the same nude rats was used to determine K_i_ by quantitative autoradiography (QAR) as described by us earlier (Asotra et al., 2003). Animals were transferred to a radiation-secure facility, placed under maintenance anesthesia, and prepared for constant blood withdrawal as described by Asotra et al. (2003). Briefly, 5 min after the start of the i.v. infusion of vehicle, NS1619 (30 μg/kg/min for 15 min), 100 μCi/kg of [^14^C] sucrose in 1 ml phosphate-buffered saline (PBS) was injected as an i.v. bolus within 15 s. Rats with abnormal blood gases or blood pressure were excluded from the study. The Ki (μl/g/min), which is an initial rate for blood-to-brain transfer of [^14^C] sucrose was calculated as described by Ohno et al. (1978). The Ki was determined for [^14^C] sucrose in the tumor core, tumor-adjacent brain tissue, and contralateral normal brain tissue.

### VALIDATION OF MRI WITH QAR

For basal BTB permeability (without any BTB modulation) measurements, nude rats were infused with PBS followed by Gd-DTPA and DCE-MR images obtained. Briefly, a bolus Gd-DTPA (0.2 mmol/kg) will be delivered within 5 s via a tail vein catheter. DCE-MRI data analysis was done via the reference region model as previously described (Provenzale et al., 2002; Yankeelov et al., 2005a, b); this analysis returns K^trans^ and v_e_. QAR with the radiotracer [^14^C] sucrose was performed as previously described (Ohno et al., 1978; Asotra et al., 2003). K_i_ was determined for four voxels each from the tumor core and tumor periphery on each slice of five contiguous QAR slices. We determined the overall K_i_ for the tumor border and tumor core by averaging the results for all five slices.

### CO-REGISTRATION

The non-invasive, DCE-MRI parameter maps measuring K^trans^ and v_e_, was co-registered with QAR images on a slice by slice basis. By this way, we could correlate both global and local changes in BTB permeability as measured by these two different techniques, and validate the use of DCE-MRI measurements. Correlation of the DCE-MRI measurements to QAR images is a novel accomplishment, offering opportunities for performing preclinical anticancer drug screening.

The linear regression curve was drawn using the K_i_ versus K^trans^ scatter plots and the regression coefficient was calculated, which indicates significance of the correlation between the two measures. A high correlation coefficient between the two different measures would demonstrate that a non-invasive, clinically relevant DCE-MRI metric of tissue vessel perfusion-permeability (as assessed by the reference region model) correlates significantly with the QAR technique. This represents a validation of the reference region model for the analysis of DCE-MRI data in, at least, the rodent glioma model that can be translated to a clinical set up.

### QUANTITATIVE AUTORADIOGRAPHY

After allowing washout of Gd-DTPA for 24 h the same rats were subjected to QAR analysis using [^14^C]-labeled sucrose. Following QAR preparation, the regional permeabilities in several tumor region of interests (ROIs) were estimated by computing the unidirectional transfer constant, Ki as described in previously published studies (Ohno et al., 1978; Asotra et al., 2003). ROI analysis was conducted on a set of four pixels from each side of the tumor rim, from four pixels within the tumor core, and four pixels from the entire tumor. These same ROIs were then selected from six contiguous sections and averaged to yield one value for Ki for the tumor rim, one value for the tumor core, and one value for the whole tumor average for each animal. These values were then used for correlation with the DCE-MRI estimate of K^trans^ described above. For comparison to the averaged QAR permeability (Ki) averaged DCE-MRI permeability (K^trans^) values were obtained for the tumor rim, tumor core, and the whole tumor. In each case, voxels were selected and the time courses from each voxel were averaged to form a single time course which was then analyzed. In particular, one voxel from each side of the tumor was selected to construct the tumor rim time course, four contiguous voxels from the tumor center were selected to produce tumor core time course, and all enhancing voxels were averaged to produce a single time course for the whole tumor.

DCE-MRI data analysis as done via the reference region model to return estimates of K^trans^ and *v*_e_ (the extravascular extracellular volume fraction) for the tumor rim, tumor core, and whole tumor average. QAR with [^14^C] labeled sucrose was performed to obtain Ki estimates for these same regions. These two measurements were then compared. A correlation analysis between DCE-MRI (K^trans^) and QAR (Ki) for the whole tumor was performed. The scatter plot and regression line was drawn.

### EFFECT OF NS-1619 ON BTB PERMEABILITY CHANGES IN C6-GLIOMA RAT

A significant number of brain tumor patients do not respond well to anticancer agents. This is most likely due to the inability of anticancer agents to cross the BTB and reach cancer cells in the brain in effective quantities. It has recently been shown that BK_Ca_ channels regulate both BTB permeability and tumor cell proliferation in rat brain tumors (Ningaraj et al., 2002, 2003a). Therefore, after validating the MRI with QAR method we investigated whether NS-1619 increases BTB permeability (K^trans^) and extravascular extracellular volume fraction (*v*_e_) because these parameters have been applied much to pathology, including brain tumors. Here we compared the permeability of C6 brain tumors in rats that received BK_Ca_ channel agonist, NS-1619 to C6 brain tumors and in rats that did not receive NS-1619.

Eight female Wistar rats (weight range, 180–200 g) were intracranially implanted with 2 × 10^5^ C6 glioma cells as described earlier, and randomized to saline + Gd-DTPA and NS-1619 + Gd-DTPA groups. We applied the same MRI protocol as described above for this investigation. Imaging and tumor models were identical with the exception that the rat in the top frame received NS-1619 immediately prior to imaging, while the rat shown in bottom frame received saline. This indicates that the blood vessels associated with tumor with treatment are more permeable and/or more highly perfuse. There is a clear difference in the enhancement (DR1) between the two groups of animals. We showed that a non-invasive, clinically relevant DCE-MRI metric of tissue vessel perfusion-permeability (as assessed by the reference region model) is sensitive to changes in blood vessel permeability following administration of NS-1619 as shown by us previously (Ningaraj et al., 2002, 2003a; Black and Ningaraj, 2006).

### STATISTICS

Data from previous studies in rat glioma model showed an increase in BTB permeability from 30.0 ± 1.5 μl/g/min (*n* = 6, mean ± SE) in control animals to 70.0 ± 6.0 μl/g/min (*n* = 6) in NS169-treated rats. Both parametric and non-parametric procedures were employed as appropriate; for the validation of MRI by QAR under the two conditions with and without modulation of potassium channels, a stratified Spearman’s rank correlation coefficient was calculated using SAS software.

### TO STUDY WHETHER MRI CONTRAST ENHANCEMENT AROUND BRAIN TUMOR EDGES CAN BE ACHIEVED BY INJECTING ATP-SENSITIVE POTASSIUM (K_**ATP**_) CHANNEL ACTIVATOR

We injected K_ATP_ channel opener minoxidil sulfate (MS) intravenously (tail vein) for selective and transient opening of BTB to study whether delivery of Gd-DTPA to leading tumor edges for enhancing contrast of diffused tumor.

## RESULTS

### VALIDATION OF MRI WITH QAR

**FIGURE 1:**
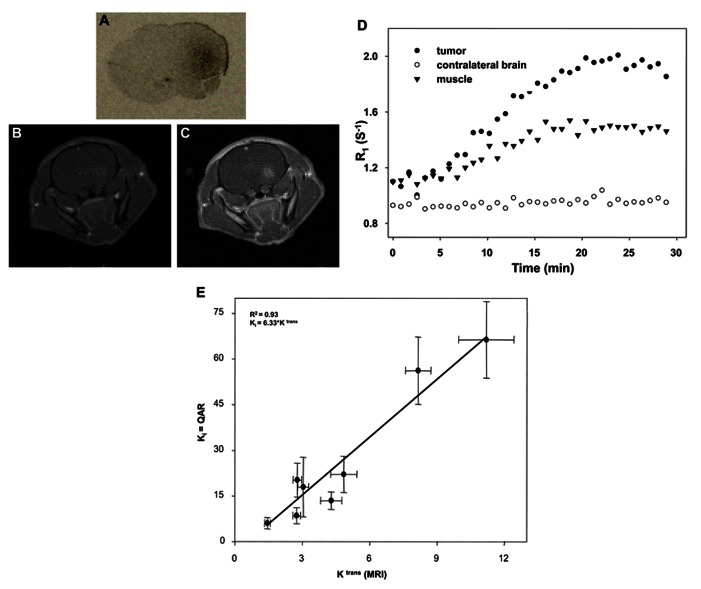
**Validation of DCE-MRI with QAR method: axial views of the tumor from a central slice of a rat, QAR image (A), pre-contrast and 10 min post-contrast MR image **(B,C)**.** The scatter plot displays the Ki and K^trans^ for the pooled data **(D)**. The *r*^2^ is 0.93 (*P* < 0.05), indicates a significant relationship between Ki and K^trans^ (Toda, 2003; Black and Ningaraj, 2004) The regression line has a slope of 6.33 **(E)**. The correlation coefficient (*r*^2^) of 0.93 with *P* < 0.05, indicates a strong and significant relationship between K_i_ and K^trans^.

A study performed in rat glioma (C6) model to correlate two distinct methods to validate MRI method with QAR method. **Figure 1** shows the axial views of the tumor from a central slice of a rat, QAR image (**Figure 1A**), pre-contrast and 10 min post-contrast MR image (**Figures 1B, C**). The scatter plot displays the Ki and K^trans^ for the pooled data. The scatter plot displays the Ki and K^trans^ for the pooled data (**Figure 1D**). The *r*^2^ of 0.93 (*P* < 0.05), indicates a significant relationship between Ki and K^trans^ (Toda, 2003; Black and Ningaraj, 2004). The regression line with a slope of 6.33 is shown (**Figure 1E**). This shows that the non-invasive, clinically relevant DCE-MRI metric of brain tissue blood vessel perfusion-permeability (as assessed by the reference region model) correlates significantly with the invasive QAR clinically irrelevant technique. This represents a validation of the reference region model for the analysis of DCE-MRI data in, at least, the C6 glioma tumor model, which could be extrapolated to human brain tumors in a clinical situation.

### NS-1619-INDUCED BTB PERMEABILITY CHANGES IN C6-GLIOMA RAT

**FIGURE 2:**
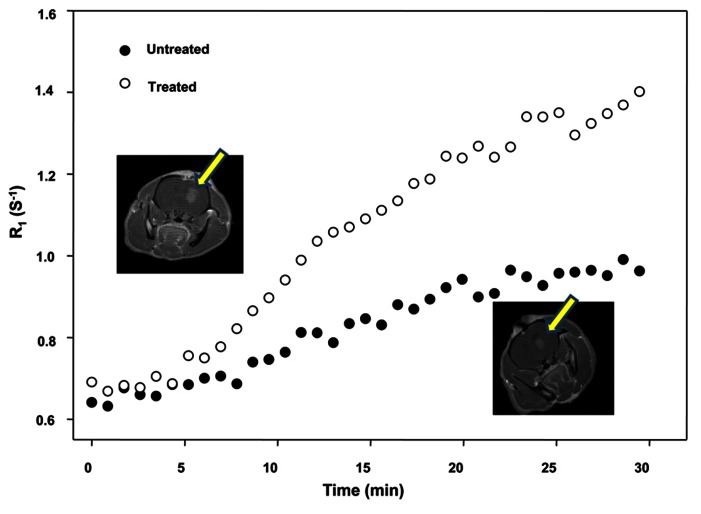
**Effect of NS-1619 on K^**trans**^ showing the increased level of contrast enhancement in the NS-1619-treated rat as quantified by DCE-MRI analysis.** The tumor volumes measured by T_1_-weighted images are approximately 38.94 and 44.10 mm^3^ for the top and bottom frames, respectively. Tumor volumes were computed by manually outlining the enhancing region of the brain (for each slice) and multiplying the number of voxels within each ROI by the voxel size (0.273 mm^3^). The increased level of enhancement in the treated rat was quantified by DCE-MRI analysis. The control group K^trans^ mean was 1.83 ± 0.59/min, while the treatment group was 9.20 ± 7.69/min; this difference is significant at the *P* < 0.05 level. The control group *v*_e_ mean was 0.16 ± 0.07, while the treatment group was 0.19 ± 0.07; this difference was not significant.

**Figure 2** shows the increased level of contrast enhancement in the NS-1619-treated rat as quantified by DCE-MRI analysis. The tumor volumes measured by T_1_-weighted images are approximately 38.94 and 44.10 mm^3^ for the top and bottom frames, respectively. Tumor volumes were computed by manually outlining the enhancing region of the brain (for each slice) and multiplying the number of voxels within each ROI by the voxel size (0.273 mm^3^). The increased level of enhancement in the treated rat was quantified by DCE-MRI analysis. The control group K^trans^ mean was 1.83 ± 0.59/min, while the treatment group was 9.20 ± 7.69/min; this difference is significant at the *P* < 0.05 level. The control group *v*_e_ mean was 0.16 ± 0.07, while the treatment group was 0.19 ± 0.07; this difference was not significant.

**FIGURE 3:**
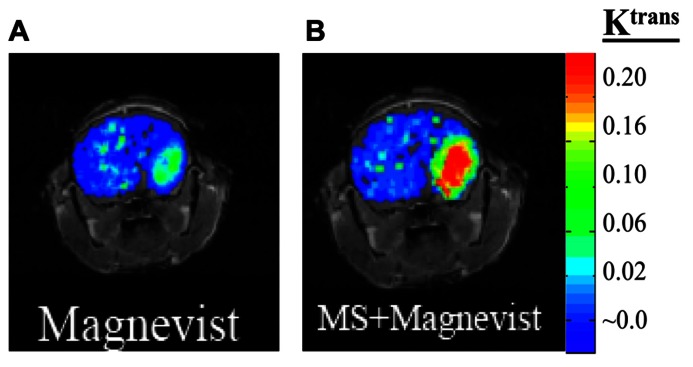
**Mouse with glioma (GL-261) was injected with 0.2 mmol/kg of MRI contrast agent Magnevist and DCE-MRI studies performed (A).** After 24 h washout period the same mouse was given ATP-sensitive potassium (K_ATP_) channel activator, minoxidil sulfate (MS) prior to Magnevist infusion **(B)**.

Magnetic resonance imaging contrast enhancement around brain tumor edges: we showed that K_ATP_ channel opener (MS) increases Gd-DTPA delivery for enhancement of tumor edges (**Figure 3**).

## DISCUSSION

Correlation analysis between DCE-MRI and QAR: the quantitative, non-invasive assessment of tumor growth and treatment response has become an increasingly important and attainable goal in oncology. Furthermore, the non-invasive measurements of tissue status have been shown to correlate with disease state, diagnosis, and treatment response. Thus, there is great and continuing interest in this methodology. While there is much interest in the applications of this method to assess tumor growth and treatment response, there has been comparatively little study of the correlation of this non-invasive technique with more accepted (i.e., “gold-standard”) measurements of tumor perfusion (Healy et al., 1987; Li et al., 2000; Tofts and Kermode, 1991; Tofts, 1997; Zhu et al., 2000). In fact, at a recent National Cancer Institute special workshop on translating DCE-MRI methodology into routine clinical use, a comment that was repeatedly made was the necessity of developing appropriate methods of validating the measurements made by DCE-MRI. In line with this, we conducted experiments to explore the relationship between the minimally invasive, clinically relevant measures of vessel perfusion offered by DCE-MRI to those of the invasive, clinically irrelevant, “gold-standard” of QAR. In particular, we compared the measurement of the QAR transfer constant Ki, to the DCE-MRI transfer constant K^trans^ in a C6 rat glioma model (**Figure 1**). Both transfer constants measure the initial rate for blood-to-brain transfer of a tracer.

### BTB PERMEABILITY MODULATION-CORRELATION OF MRI AND QAR

We showed that a non-invasive, clinically relevant DCE-MRI metric of tissue vessel perfusion-permeability (**Figure 2** as assessed by the reference region model) is sensitive to changes in blood vessel permeability following administration of BK_Ca_ channel activator NS-1619 (Ningaraj et al., 2002; Black and Ningaraj, 2006). We have shown that human gliomas and brain tumor ECs overexpress potassium channels that can be targeted for increased BTB permeability (Ningaraj et al., 2002, 2003b, 2009a; Black and Ningaraj, 2006). It is anticipated that these experiments provide a basis for targeting ECs that overexpress potassium channels with specific activators to permit increased MRI enhancing agent delivery selectively to brain tumors. Monitoring the outcome of increased MRI enhancing agents’ delivery to microsatellites and leading tumor edges in glioma patients would lead to beneficial clinical outcome.

Our findings may have significant impact on CNS drug delivery by shedding light on the function of the BTB that can be used to develop novel drug delivery approaches for molecular-targeted therapy, and further develop methods for non-invasively imaging BTB permeability changes and the response of glioma to treatment. Validated MRI permeability measurement technique may allow patient-specific therapy to improve clinical outcome in patients with gliomas. Our research is expected to offer an early and important translational component to the clinical practice. When a patient is diagnosed with glioma, definitively ruling out the presence or absence of additional lesions in brain is important for diagnosis and for deciding upon possible surgical management. BTB permeability modulation following administration of potassium channel activators will significantly increase the delivery of Magnevist for greater enhancement of leading tumor edges as shown in **Figure 3**, and potentially microsatellites using DCE-MRI. Furthermore, use of targeted therapies will hopefully lead to better treatments for this deadly disease. Our present research aims to develop methods to increase anticancer drug delivery selectively to glioma cells and retain the drug longer in tumor cells so that toxicity in the normal brain is prevented. In addition, the early proteomic response data before and after anticancer treatment would provide valuable insights on differentiating responders from non-responders based on early tumor response to a drug regimen. This will augment decisional matrix of clinicians and patients, and spare patients from unnecessary side effects of cytotoxic drugs. Nevertheless, further studies are required to validate the DCE-MRI by co-registering basal and modified (increased) permeability measurements obtained by DCE-MRI and QAR measurements in human glioma models. This provides a non-invasive means to measure subtle BTB leakage associated with glioma growth. To visualize the invasive and diffused glioma for effective treatment, including surgery, the MRI agents should be efficiently delivered to the tumor edges. In this regard, we showed in a mouse glioma xenograft, the biochemical modulation of BTB with MS increases Magnevist delivery to tumor edges (**Figure 3**).

Enhancing therapeutic efficacy for heterogeneous and aggressive tumors such as gliomas can be achieved by attacking the cancer cells at different mechanistic pathways simultaneously. In controlled experiments involving mouse and rat brain tumor xenografts, we determined the optimal dose and time of administration of potassium channel activators (NS1619 and MS). These activators were found to be non-toxic to animals when evaluated in human brain tumor xenograft models (Ningaraj et al., 2002, 2003b, 2009a). In our laboratory, the studies are in progress by employing DCE-MRI to detect and measure microsatellites and diffused glioma edges in normal brain following transient potassium channel activator-induced BTB permeability increase to Gd-DTPA. In addition, we are investigating the effect of *KCNMA1* (codes for alpha subunit of BK_Ca_ channels) down-regulation on BTB permeability in human brain tumor model. We seek to demonstrate whether tumor developed using U87 MG cells with *KCNMA1* down-regulation fail to elicit similar BTB permeability increase to Gd-DTPA following NS169 infusion compared to that of the wild type tumor. Since we have already validated the DCE-MRI with QAR BTB permeability measurements, now work is underway to quantitatively measure the BTB permeability in metastatic brain tumor models developed with intracranial injection of breast and lung metastatic cancer cell lines with and without *KCNMA1* knockdown. With this strategy, we seek to study the role of *KCNMA1* in BTB permeability regulation in human brain tumor xenograft models.

In conclusion, it is anticipated that this research will provide a basis for targeting primary and metastatic brain cancer cells and the BTB that overexpress potassium channels with agents that activate potassium channels. Thus we might increase penetration of anticancer drugs and MRI contrast enhancing agents selectively to brain tumors in patients resulting in beneficial clinical outcomes.

## Conflict of Interest Statement

The authors declare that the research was conducted in the absence of any commercial or financial relationships that could be construed as a potential conflict of interest.
